# TRANSLATING DATA INTO ACTION ON BRAIN HEALTH

**DOI:** 10.1093/geroni/igae098.1555

**Published:** 2024-12-31

**Authors:** John Shean, Erin Bouldin, John Shean

**Affiliations:** Chair; Co-Chair; Discussant

## Abstract

The Healthy Brain Initiative (HBI) is a collaborative effort to advance brain health as central to public health in the US. Fundamental to the HBI effort is the HBI Road Map Series, frameworks that help state and local public health departments prioritize actions to address brain health across the life course and support caregivers. Data analysis and utilization are key tenets of the Road Maps Series and are essential to educate the public and to inform policy and planning. In 2023, the Alzheimer’s Association piloted Data for Action — a new HBI project to support states in utilizing their existing datasets on brain health and caregiving. This symposium highlights the process, successes and lessons learned from the Data for Action pilot. The first presentation will provide an overview of data available from the Behavioral Risk Factor Surveillance System Cognitive Decline and Caregiving modules. The second presentation will describe the development and structure of the Data for Action pilot project, including the HBI and Road Map Series. In the third presentation, Arkansas, one of the pilot states, will present their process and products from Data for Action. The fourth presentation will summarize the products and impacts from the other states involved in the pilot and will discuss best practices and lessons learned from the project. A discussant will help participants understand how, through collaborative action, brain health and caregiving data can be harnessed to drive decisions, policies, and action at the state level.

